# Similarity of molecular phenotype between known epilepsy gene LGI1 and disease candidate gene LGI2

**DOI:** 10.1186/1471-2091-11-39

**Published:** 2010-09-24

**Authors:** Vachiranee Limviphuvadh, Ling Ling Chua, Rabi 'Atul' Adawiyah Bte Rahim, Frank Eisenhaber, Sebastian Maurer-Stroh, Sharmila Adhikari

**Affiliations:** 1 Bioinformatics Institute, (BII), Agency for Science, Technology and Research (A*STAR), 30 Biopolis Street, #07-01 Matrix, 138671, Singapore; 2 Department of Biological Sciences, National University of Singapore (NUS), 14 Science Drive 4, 117543, Singapore; 3 School of Computer Engineering (SCE), Nanyang Technological University (NTU), 50 Nanyang Drive, 637553, Singapore; 4 School of Biological Sciences (SBS), Nanyang Technological University (NTU), 60 Nanyang Drive, 637551, Singapore

## Abstract

**Background:**

The LGI2 (leucine-rich, glioma inactivated 2) gene, a prime candidate for partial epilepsy with pericentral spikes, belongs to a family encoding secreted, beta-propeller domain proteins with EPTP/EAR epilepsy-associated repeats. In another family member, LGI1 (leucine-rich, glioma inactivated 1) mutations are responsible for autosomal dominant lateral temporal epilepsy (ADLTE). Because a few LGI1 disease mutations described in the literature cause secretion failure, we experimentally analyzed the secretion efficiency and subcellular localization of several LGI1 and LGI2 mutant proteins corresponding to observed non-synonymous single nucleotide polymorphisms (nsSNPs) affecting the signal peptide, the leucine-rich repeats and the EAR propeller.

**Results:**

Mapping of disease-causing mutations in the EAR domain region onto a 3D-structure model shows that many of these mutations co-localize at an evolutionary conserved surface region of the propeller. We find that wild-type LGI2 is secreted to the extracellular medium in glycosylated form similarly to LGI1, whereas several mutant proteins tested in this study are secretion-deficient and accumulate in the endoplasmic reticulum. Interestingly, mutations at structurally homologous positions in the EAR domain have the same effect on secretion in LGI1 and LGI2.

**Conclusions:**

This similarity of experimental mislocalization phenotypes for mutations at homologous positions of LGI2 and the established epilepsy gene LGI1 suggests that both genes share a potentially common molecular pathogenesis mechanism that might be the reason for genotypically distinct but phenotypically related forms of epilepsy.

## Background

The leucine-rich glioma inactivated (LGI/Epitempin) protein consists of an N-terminal region which has a putative signal peptide suggesting that the protein is either secreted or membrane bound followed by leucine-rich repeats (LRRs) which are flanked by cysteine-rich repeats domains [1,2]. The C-terminal region has seven EPTP repeats which form a seven bladed beta-propeller structure [2,3]. The EPTP repeats were also found in three paralogues of LGI1 (LGI2, LGI3 and LGI4) as well as in two other genes (C21orf29/TNEP1 and GPR98/VLGR1) which constitute the EPTP superfamily [3,4].

LGI1 is the best studied gene of the LGI family and it is responsible for causing autosomal dominant lateral temporal epilepsy (ADLTE) or autosomal dominant partial epilepsy with auditory features (ADPEAF), one type of familial temporal lobe epilepsy. It was the first human idiopathic epilepsy known to be caused by mutations in a non-ion channel gene [5-7]. Epilepsy is a brain disorder in which clusters of nerve cells, or neurons, in the brain sometimes signal abnormally. Several studies have been carried out on LGI1 and had shown that point mutations on this gene have caused defects in secretion [8-10]. Also, clinical studies have shown that up to 50% of patients with ADTLE have mutations in their LGI1 gene [11,12]. Using gene-history and expression analyses, LGI2-4 have been suggested as candidate genes for human disorders [13].

In our previous work, arguments based on protein sequence analysis and patient-specific chromosomal deletions are provided for LGI2 as the prime candidate gene for partial epilepsy with pericentral spikes (PEPS) [14]. Both PEPS and ADLTE are temporal lobe epilepsies and LGI1 and LGI2 are closely related paralogues that share the same functional domains. The overall aim of the study is to investigate whether SNPs in LGI2 have similar effects as in LGI1 by causing secretory defects, the known pathogenic mechanism of several LGI1 mutations [8-10]. Thus, we experimentally investigated both the reported but yet functionally uncharacterized mutations and SNPs in LGI1 and LGI2 genes, as well as a few additional mutations with theoretically derived functional hypothesis. We observed a reproducible phenotype in terms of lack of protein secretion (resulting in loss of function) for both LGI1 and LGI2 if structurally homologous positions are mutated that are conserved throughout the LGI family and known to cause disease in LGI1. Hence, we suggest that there is a similar underlying disease mechanism for LGI1 and LGI2 and we propose that each of the LGI family members might be responsible for phenotypically similar, mechanistically related but genotypically distinct forms of epilepsy.

## Results

### Mapping of non-synonymous SNPs and known disease mutations

**Figure 1:**
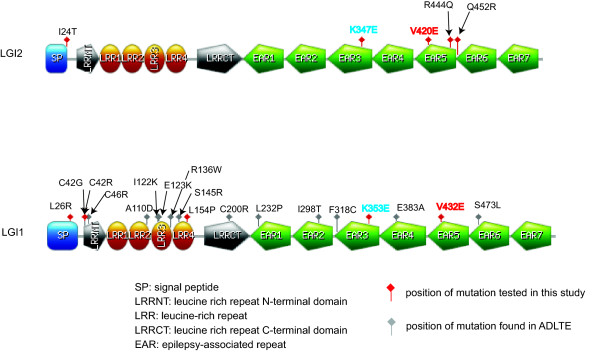
**Domain architectures of LGI2 and LGI1**. We annotated four SNPs of LGI2, 16 ADLTE missense mutations found in LGI1 (and the artificial LGI1 L26R) and two additional mutations (LGI1 K353E and LGI2 V420E) constructed in this study. Mutations are indicated as lollipops (red for mutations experimentally tested in this study and grey otherwise).

We searched for non-synonymous SNPs and known disease mutations of LGI1 and LGI2 in the literature (PubMed) [10] and dbSNP [15] and mapped them to the domain architectures of LGI1 and LGI2 (Figure 1). In total, we found data for 16 missense mutations for LGI1 that cause ADLTE and 4 non-synonymous SNPs for LGI2 (without reported phenotype). Six of the LGI1 mutations (L232P, I298T, F318C, E383A, V432E and S473L), as well as 3 out of the 4 SNPs in LGI2 (K347E, R444Q and Q452R), were localized in the EAR propeller region that presumably interacts with ADAM metalloproteases [16]. Moreover, one of the SNPs in LGI2 (I24T) is found in the N-terminal region comprising the signal peptide. Therefore, we put an artificial mutation LGI1 L26R [17] which is located in the signal peptide region for additional study concurrently with LGI2 I24T. The remaining mutations in LGI1 are spread throughout the N-terminal half that includes the leucine-rich repeats.

### Analysis of severity of the mutations in the evolutionary context

We investigated all SNPs and missense mutations of LGI1 and LGI2 in the evolutionary context of the alignment of LGI family members by using SIFT [18] and PolyPhen [19]. These programs predict the severity of effects of a mutation depending on the degree of amino acid conservation in the mutated position and how different, in terms of physicochemical properties, a mutated residue is compared to the repertoire of amino acids naturally found in this position.

The summary of results of SIFT and PolyPhen are shown in Table 1. For SIFT, all 16 missense mutations of LGI1 gave a score lower than 0.05. This implies they are predicted to be deleterious and affect protein functions. PolyPhen predicts that most of the missense mutations are probably damaging (only the E123K and I298T mutations are predicted to be only possibly damaging). For the artificial mutation LGI1 L26R, SIFT and PolyPhen results are predicted to be tolerated and benign, respectively. Among the 4 non-synonymous SNPs of LGI2, only K347E is predicted by SIFT to be deleterious. I24T and R444Q have scores close to the classification threshold (0.05). Similarly, only K347E is predicted by PolyPhen to be possibly damaging, while the other three are all predicted to be benign.

**Table 1:** Summary of results from SIFT, PolyPhen, secretion and subcellular localization studies.

	Mutation^	Position	SIFT result, score	Polyphen result, score difference	Secretion studies	Subcellular localization studies
**LGI2**	I24T	SP	Tolerated, 0.06	Benign, 1.125	-*	-*
	K347E	EAR	Deleterious, 0	Possibly damaging, 1.903	-*	-*
	R444Q	EAR	Tolerated, 0.07	Benign, 1.342	-*	-*
	Q452R	EAR	Tolerated, 0.63	Benign, 0.366	-*	-*
	V420E (corresponds to LGI1 V432E)	EAR	Deleterious, 0	Probably damaging, 2.173	+*	ER-retention*

**LGI1**	L26R (artificial mutation)	SP	Tolerated, 0.34	Benign, 1.350	+*	ER-retention*
	**C42G**	LRRNT	Deleterious, 0	Probably damaging, 2.700	N/A	N/A
	**C42R**	LRRNT	Deleterious, 0	Probably damaging, 2.700	+*	ER-retention*
	**C46R**	LRRNT	Deleterious, 0	Probably damaging, 3.833	**+**[8]	N/A
	**A110D**	LRR	Deleterious, 0	Probably damaging, 2.026	+ [10]	N/A
	**I122K**	LRR	Deleterious, 0	Probably damaging, 2.741	**+ **[47]	N/A
	**E123K**	LRR	Deleterious, 0	Possibly damaging, 1.841	N/A	N/A
	**R136W**	LRR	Deleterious, 0	Probably damaging, 2.877	**+ **[10]	N/A
	**S145R**	LRR	Deleterious, 0	Probably damaging, 2.174	**+ **[8]	N/A
	**L154P**	LRR	Deleterious, 0	Probably damaging, 2.467	+*	ER-retention*
	**C200R**	LRRCT	Deleterious, 0	Probably damaging, 3.833	**+ **[8]	N/A
	**L232P**	EAR	Deleterious, 0	Probably damaging, 2.052	**+ **[9]	N/A
	**I298T**	EAR	Deleterious, 0.01	Possibly damaging, 1.910	N/A	N/A
	**F318C**	EAR	Deleterious, 0	Probably damaging, 2.419	**+ **[8]	ER-retention [8]
	**E383A**	EAR	Deleterious, 0	Probably damaging, 2.249	**+ **[8]	ER-retention [8]
	**V432E**	EAR	Deleterious, 0	Probably damaging, 2.173	+*	ER-retention*
	**S473L**	EAR	Deleterious, 0	Probably damaging, 2.399	N/A	N/A
	K353E (corresponds to LGI2 K347E)	EAR	Deleterious, 0	Possibly damaging, 1.852	-*	-*

SIFT: Sorting Intolerant From Tolerant, PolyPhen: prediction of functional effect of human nsSNPs

SP: signal peptide, LRRNT: leucine rich repeat N-terminal domain, LRR: leucine-rich repeat,

LRRCT: leucine rich repeat C-terminal domain, EAR: epilepsy-associated repeat

**^ **mutation in bold letter means missense mutation found in ADLTE, numbering corresponds to Genbank entries NP_005088.1 for LGI1 and NP_060646.2 for LGI2

**+ **affect secretion, **- **does not affect secretion or subcellular localization, * determined in this study

Unfortunately, none of the reported mutations in LGI1 and LGI2 correspond to one and the same homologous position in the respective sequences. For later experimental study, we include two more control mutations that are structurally homologous in LGI1 and LGI2 and would map to a reported disease mutation in LGI1 or a non-synonymous SNP in LGI2. These are LGI2 V420E (corresponding to the known V432E disease mutation in LGI1) and LGI1 K353E (corresponding to the nsSNP K347E in LGI2). The SIFT method predicts for both new mutations to be deleterious and, for PolyPhen, LGI2 V420E is predicted to be probably damaging while LGI1 K353E is predicted to have a less severe effect and only be possibly damaging.

### Effects of the artificial mutation LGI1 L26R and LGI2 SNP I24T on signal peptide and cleavage site prediction

LGI1 L26R and LGI2 I24T, that are both predicted by SIFT and PolyPhen to cause little damage, are actually part of the N-terminal signal peptides that are required to translocate the proteins to the ER and out of the cell. Therefore, we analyzed if the mutations would possibly alter the localization motif as predicted by SignalP [20].

For the wild-type of LGI2, the cleavage site is predicted between positions 22 and 23 (AAC-LI) while the I24T SNP appears to still have a valid signal peptide but with a predicted cleavage site shift (positions 28 and 29 between RSA-QV). For wild-type LGI1, the cleavage site is predicted between positions 34 and 35 (TEG-KK). For the artificial mutation LGI1 L26R, SignalP does not predict a signal peptide anymore (the maximum Y-score decreases from 0.611 in the wild-type to 0.397).

As opposed to LGI2 I24T that is predicted to only have a minor effect, LGI1 L26R is in the hydrophobic helical segment of the signal peptide motif and exchange of a hydrophobic leucine with a positively charged arginine definitely alters the physicochemical properties of the whole region. Consequently, the LGI1 L26R mutation is hypothesized to alter efficiency of secretion. To clarify these predictions, we measured the effect of these mutations on secretion from mammalian cells (see below).

### Phylogenetic analysis of EAR domain containing protein families

It should be emphasized that LGI proteins have considerable sequence identity and similarity. Their alignments as well as the construction of phylogenetic tree are not difficult. In terms of pairwise sequence identity, LGI1 and LGI2 are most similar to each other (54%), while LGI4 would appear most distant to all other LGIs (47% with LGI1, 39% with LGI2 and 40% with LGI3).

**Figure 2:**
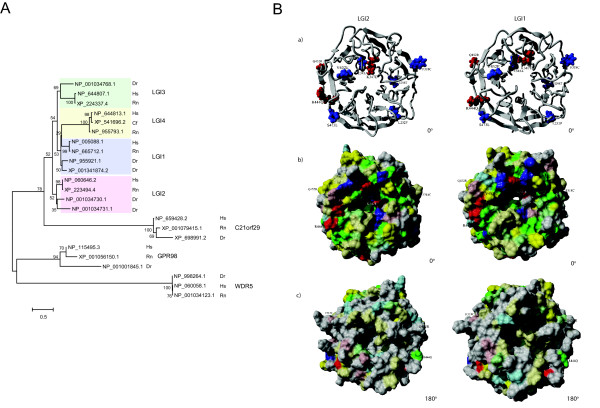
**Phylogenetic analysis of the EAR repeats containing proteins and structural model analysis of the EAR domain**. **A) Phylogenetic tree of the representative EAR repeats containing proteins**. The EAR regions of LGI family, C21orf29, GPR98 of representative species were aligned with MAFFT v6.240 using the L-INS-I algorithm and then the Neighbor-Joining method was used to construct the phylogenetic tree (see Method section for details). WDR5 is used as out-group. Phylogenetic analyses were conducted in MEGA4. NCBI accession numbers of all used sequences are shown. Species are abbreviated as follows: Hs ... Homo sapiens, Rn ... Rattus norvegicus, Dr ... Danio rerio and Cf ... Canis familiaris. **B) Structural model and conservation mapping of the EAR domain**. A) Shows the propeller domains of LGI2 and LGI1 with residues involved in SNPs of LGI2 (red colored) and mutations of ADLTE found in LGI1 (blue colored). B) Shows the conservation within the whole LGI family mapped to the surface of the propeller domain (see Method section for details) in the same orientation as in a), while c) is rotated by 180 degree to show the other side of the domain. Grey color means no conservation, while the other colors signify conservation of physical properties, i.e., yellow: hydrophobic, green: uncharged polar, blue: positive charge, red: negative charge. Color intensity is proportional to strength of conservation see Method section for more details).

Figure 2A shows the phylogenetic relationship of all known protein families containing EAR domains based on the alignment of their EAR region and using WDR5, a distantly related seven-bladed beta-propeller with resolved crystal structure, as outgroup. Among LGI family members, LGI1, LGI2 and LGI3 cluster as separate groups of paralogues that have split from each other before the separation of fish and mammals, whereas LGI4 that lacks obvious orthologues in fish appears to have diverged from the LGI1 line at an evolutionary later time point. LGI1 and LGI2 appear to have two paralogues each in zebrafish due to an additional fish-specific duplication event. The families of GPR98 and C21orf29 also cluster as separate subfamilies that diverged from each other and from the LGIs before the duplication events in the LGI family. In summary, this analysis emphasizes the closeness of LGI family members and suggests related functions.

### Mutations in LGI2 and LGI1 propeller domain structure models

Since many of the SNPs and mutations analyzed in this study are located in the EAR domains of LGI1 and LGI2 and this region is presumed to harbor the site for interaction with ADAM metalloproteases [16], we constructed a 3 D structural model of this domain (Figure 2B, see Methods section for details). The evolutionary conserved residues found in the alignment of LGI family members were mapped to the surface of the model. Obviously, there is a concentration of these residues at one side of the propeller. Indeed, beta-propeller structures often function as protein interaction modules and there are known examples where conserved sites are limited to one "flat" side of the propeller and serve as protein binding interface [21]. Hence, also the conservation pattern in our LGI2 model could be explained by having a conserved protein interaction site at this side of the propeller. This site is shared among the LGI family members (Figure 2B) and might be responsible for the interaction with ADAM metalloproteases or, possibly, other functional critical proteins. In support of this, it was shown recently that at least LGI1 and LGI4 share the same repertoire of ADAMs as binding partners [22]. Interestingly, many reported EAR domain mutations of both LGI2 (K347E and R444Q) and LGI1 (F318C, V432E and S473L) are co-localized in the conserved surface patches and, hence, are hypothesized to interfere with protein function by affecting the interaction site. For example, the K to E mutation at the position 347 of LGI2 alters the electrostatic potential of its environment. Two other mutations in the propeller domain of LGI2 (R444Q and Q452R) are not involved in the putative binding interface and could be less severe since they maintain polar physicochemical properties and they are pointing into the (polar) solvent.

### Expression patterns of LGI family members

Given the shared domain architecture among the LGI family and the conserved set of interaction partners (at least for LGI1 and LGI4 [22]), LGI family members can be assumed to have quite similar molecular functions. However, since disease causing mutations in one LGI cannot be compensated by the remaining unmutated LGIs, it has been suggested that each LGI family member might execute its respective function in different expression contexts [8,22]. In Table 2, we compile available expression data from multiple sources [23-26], and confirm that LGI family members have tissue-specific and only weakly overlapping expression patterns in different parts of the brain.

**Table 2:** Summary of expression of LGI1, LGI2, LGI3 and LGI4.

Gene	**BioGPS **[23] Human GeneAtlas U133A.gcrma experiment	**BioGPS **[23] Mouse GeneAtlas GNF1 M.gcrma experiment	**HPRD **[24] (site of expression, selected only human brain regions)	**Human Protein Atlas **[25] (selected only strong and moderate expression in human brain tissues and cell lines)	**SOURCE **[26] (only top 5*)
**LGI1**	Most expressed in amygdala, prefrontal cortex, fetal brain, hypothalamus, caudate nucleus, cingulated cortex	Highly expressed in amygdala, hippocampus, dorsal striatum, cerebral cortex, frontal cortex and cortex, respectively	Brain, nervous system	-	Brain, ganglia, umbilical cord, embryonic tissue, nerve

**LGI2**	Expressed at levels around the median value in any tissue of the brain with a slight elevation in the olfactory bulb	Highly expressed in lower spinal cord with intermediate but elevated expression in upper spinal cord, trigeminal ganglion, substantia nigra and dorsal root ganglion	Amygdala, brain, caudate nucleus, cerebellum, corpus callosum, hippocampus, spinal cord, substantia nigra, subthalamic nucleus, thalamus	cerebellum(purkinje cells; strong, cells in granular layer; moderate, cells in molecular layer; moderate) Hippocampus (neuronal cells; moderate) Lateral ventricle (neuronal cells; moderate) D341 Med (Medulloblastoma cell line), SH-SY5Y (Metastatic neuroblastoma, clonal subline of neuroepithelioma cell line SK-N-SH)	Parathyroid, embryonic tissue, mixed, unclassified, spleen (brain is ranked at 15)

**LGI3**	Specifically expressed in prefrontal cortex	Highest levels in lower and upper spinal cord as well as substantia nigra	Brain	-	Nerve, skin, brain, prostate, pancreas

**LGI4**	Generally low but constant expression in tissues of the nervous system with slightly elevated levels in dorsal root ganglion	Most highly expressed in dorsal root ganglion, trigeminal ganglion and cerebellum respectively	Brain	-	Ganglia, pharynx, nerve, ear, eye

BioGPS, SOURCE = gene expression, HPRD = protein and/or mRNA expression, Human Protein Atlas = protein expression

* Top 5 were selected from normalized expression distribution for tissue source

### Effects of LGI1 and LGI2 mutations on protein secretion

**Figure 3:**
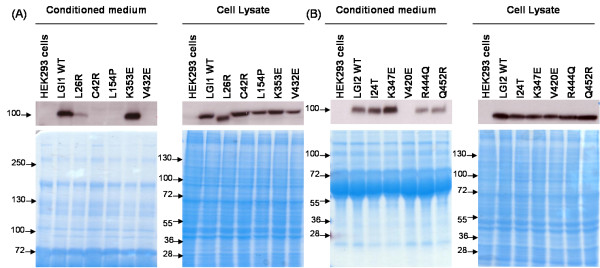
**Analysis of the effect on the secretion of A) LGI1 mutants B) LGI2 mutants**. As detailed in the Methods section, HEK293 cells were transfected with the indicated constructs and cell lysates and concentrated culture media were analysed by western blotting with an anti-GFP antibody 17-18 hours post-transfection. Simplyblue™safe staining is shown to demonstrate that all conditioned media samples were loaded equally and that all cell lysate samples were loaded similar to each other.

All SNPs in LGI2 and several not yet experimentally characterized mutations in LGI1 including the artificial mutation were tested for their effect on secretion of the protein. HEK293 cells were transfected with C-terminal GFP tagged expression constructs of wild-type or mutant LGI1 and LGI2 proteins. HEK293 cells without any expression construct were used as a control for transfection efficiency. The results are shown in Figure 3.

LGI1 WT is being secreted strongly into the conditioned medium (extracellular space); yet, some is being retained in the lysate. Only with long exposure time, the artificial L26R mutant can still be detected in the conditioned medium, which means that LGI1 L26R is just minimally secreted. The EAR domain mutation K353E in LGI1 (as well as the mutation K347E in LGI2 at the homologous sequence position) does not seem to have any effect on the secretion of the protein as compared to the other mutations of LGI1. In all other cases, the LGI1 mutation constructs resulted in robust LGI1 expression in cell lysates indicating that the mutations in LGI1 result in secretory defects. Also, the secreted protein of LGI1 WT, LGI1 L26R and LGI1 K353E were observed to be of higher molecular weight as compared to those that are being retained in the lysate suggesting posttranslational modifications of the former along the secretion pathway (Figure 3A).

On the other hand, mutations in LGI2 (with the exception of LGI2 V420E) did not show any change in the secretion level when compared to that of the wild-type. The LGI2 protein was observed both in the conditioned media and cell lysate indicating that most SNPs in LGI2 do not cause secretory defects. However, LGI2 V420E was found to be retained in the lysate similar to LGI1 V432E where the mutation is in the homologous sequence position. Similarly, secreted proteins of LGI2 were observed to be of higher molecular weight as compared to those being retained in the lysate (Figure 3B).

### Glycosylation of LGI proteins

**Figure 4:**
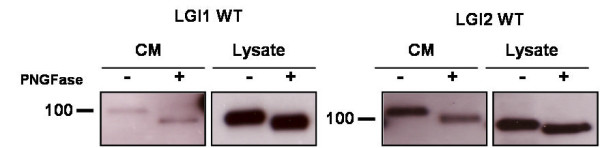
**Glycosylation of LGI1 and LGI2 mutations**. HEK 293 cells were transfected with LGI1WT and LGI2 WT constructs respectively, harvested 17-18 hours post transfection, lysed and subjected to treatment in the absence (-) or presence (+) of PNGFase. The gel shifts indicate glycosylation events.

The expected protein band size is about 92 kDa but the actual band size is almost 100 kDa. The higher mass observed might also be due to glycosylation or other modifications of the protein [8]. To test this, we performed PNGase F treatment of LGI1 WT and LGI2 WT. Figure 4 shows that both isoforms undergo a substantial molecular weight shift when treated with PNGase F, suggesting that both are indeed glycosylated.

### Subcellular localisation of LGI1 and LGI2 mutant proteins

**Figure 5:**
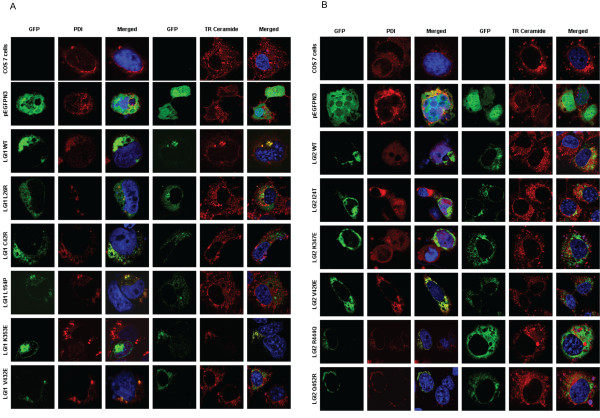
**Subcellular localization of A) LGI1 and B) LGI2 GFP-tagged wild-type and mutant proteins**. COS7 cells were transfected with GFP-fused protein (green) as indicated and treated with either an anti-PDI followed by alexa 546 (red) to stain the endoplasmic reticulum or TR Ceramide to detect the Golgi apparatus and DAPI (blue) to stain the nuclei and then examined by laser fluorescence confocal microscopy. The fields shown were visualized independently at the appropriate wavelength for GFP (488 nm) and anti-PDI or TR Ceramide (546 nm), and then the two images were merged. Magnification: 63×. Scale bar is 10 μm.

With microscopy, the subcellular localization of the non-secreted mutant variants was studied. COS7 cells were transfected with expression constructs for each mutation including a C-terminal GFP-tag based on the pEGFPN3 vector. COS7 cells with the empty vector were used as controls. We found that LGI1 WT and LGI1 K353E are localized predominantly in the Golgi and partially in the ER. But the other mutants are more predominant in the ER (Figure 5A).

As for LGI2 and its mutants, they show similar localization in both ER and Golgi with the exception of LGI2 V420E which is localized mainly in the ER (Figure 5B). These observations are consistent with the secretory western blot results as proteins that are secreted most likely are translated and processed in the rough ER and transported to the Golgi vesicles before they are being secreted. As for proteins that have secretory defects, they are most likely retained in the ER because they are misfolded or otherwise not normally processed because of their mutations.

## Discussion

In this study, we report that LGI disease mutations alter protein secretion and stability showing that the clinical epilepsy phenotype likely arises from loss of protein function. This is in agreement with LGI1 knockout mice that showed lethal epileptic seizures [27,28]. While experimental data for LGI1 is abundant, LGI2 has been mostly neglected so far. This is the first report to show effects of LGI2 SNPs in detail.

Previous and this work show that molecular pathogenesis of several LGI1 mutations involves alteration of secretion levels of LGI1 which causes the protein to be retained in the cells and, thereby, abolishes its potential to function in its normal signaling role in the extracellular space of the synaptic cleft [8,29]. We explored the effect of secretion of several LGI1 and LGI2 mutations and our data strongly suggest that mutations at sequentially and structurally homologous positions have the same effect on secretion in LGI1 and LGI2.

Protein secretion via the standard secretion pathway requires a functional signal peptide as targeting motif. We found one signal peptide related SNP in LGI2 and we studied the artificial mutation LGI1 L26R which is also located in the signal peptide region. We demonstrated that LGI1 L26R is secreted to a much lesser extent compared to wild-type LGI1. We show by PNGase F treatment that, upon secretion, both LGI1 and LGI2 are glycosylated, which has not been shown previously for LGI2.

Our study increases the total number of mutations in LGI1 and LGI2 that were tested experimentally for effects on secretion from previously 9 to 19. Interestingly, the computational classification of the mutations by SIFT [18] and Polyphen [19] as tolerated or deleterious coincides with the observation of normal or lack of secretion, respectively, in 16 out of 19 cases (see Table 1). The only obviously wrong prediction is associated with the artificial L26R mutation in the signal peptide, a non-globular segment. Tools like SIFT and Polyphen have been developed for learning set of mutations in globular 3 D structures and can provide a reasonably correct estimate regarding disease effects if new mutations in globular regions of LGI family members need to be tested.

However, when all but 1 SNP are outside of the signal peptide motif, how can they influence secretion? SIFT and Polyphen evaluated the alteration of amino acid properties following the mutation at otherwise evolutionary conserved positions and its role for the intactness of the 3 D structure of globular domains. We found a possible answer by experimentally investigating the whereabouts of the non-secreted mutants in the cell using a fluorescent label that allows us to visualize the protein's subcellular localization. We observe that proteins without secretion phenotype, including the wild-type, are predominantly found in the Golgi (before leaving the cell) while the mutants that are known to cause disease (in the case of LGI1) cannot leave the cell and are accumulated in the ER. This can be interpreted as classical fate of misfolded proteins that are caught and degraded by quality control mechanisms in the ER that ensure that only properly folded proteins are allowed to move further along the secretory pathway [30]. Our results are in agreement with previous studies for other missense mutations of LGI1 that were also found to be defective in secretion and to accumulate in the ER [8-10,17].

Finally, in order to test our hypothesis that LGI2 could follow a similar disease mechanism as LGI1 we introduced new mutations of selected residues that are (A) part of the conserved EAR propeller because it is presumably functionally important, (B) structurally homologous in LGI1 and LGI2 and (C) tested as mutations in their respective proteins for secretion phenotypes. In particular, we introduced the same mutation that caused secretion deficiency in LGI1 (V432E) to LGI2 (V420E) and also observed lack of secretion of the LGI2 mutant. Inversely, a mutation that did not alter secretion in LGI2 (K347E) also did not affect LGI1 (K353E). These results finally corroborated the similarity in mutational effects on secretion between LGI1 and LGI2.

On the other hand, LGI2 K347E and LGI1 K353E are predicted to be deleterious by both SIFT and PolyPhen, apparently, because of the charge reversal but they did not show any ER retention or secretion deficiency and, thus, appear to fold properly. LGI2 K347E and LGI1 K353E are structurally homologous and located centrally in an evolutionary conserved region on the surface of the EAR propeller structure and it is unlikely that the charge conservation is not important for function. If the conserved surface patch at the one side of the EAR propeller is indeed the critical site for interactions (e.g., with ADAM metalloproteases), then it is likely that the pathogenetic mechanism is related to an inhibition of physiological interactions.

Recently, the pattern of expression of each LGI gene was studied using digoxigenin-based *in situ *hybridization. LGI family members demonstrated distinct expression in different regions of the brain [5,29,31,32]. LGI1 is intensely expressed in intrahippocampal circuitry consistent with the clinical temporal lobe epilepsy syndrome associated with mutations of LGI1 [5,29,32]. On the contrary, the distribution of LGI2 and LGI4 expression is confined to the medial septum and nucleus reticularis thalami. Finally, the LGI3 gene exhibits a widespread expression in different neuronal types [32]. These new results, as well as our collection of previous expression data (Table 2) suggest that specialization of the individual LGI subfamily members is achieved by expression in different tissues or cell types. This also explains why disease mutations in LGI1 are not rescued by the other LGIs despite their high sequence similarity.

Experimental testing of cellular effects of mutations in both LGI1 and LGI2 reported in this work results in an effective doubling of experimentally characterized mutations in LGI proteins and, hence, generally advances our understanding of molecular disease mechanisms of LGI-related epilepsies. Following the line of evidence presented in this paper and collected from previous reports, we arrive at the conclusion that LGI1 and LGI2 apparently share the cellular mechanism of causing genotypically distinct but phenotypically related forms of epilepsy. The multiplicity of functionally redundant LGI genes appears necessary due to the hardly overlapping tissue-specific expression patterns.

## Conclusions

We provide substantial arguments for LGI2 as candidate for epilepsy phenotypes compatible with PEPS. Furthermore, we find similarity of experimental mislocalization phenotypes for mutations at homologous positions of LGI2 and the established epilepsy gene LGI1, suggesting that both genes share a common molecular disease mechanism causing genotypically distinct but phenotypically related forms of epilepsy.

## Methods

### Multiple alignment and phylogenetic tree of EAR propeller domains

For the multiple alignment of the LGI family, orthologues for each LGI subfamily were downloaded from OMA [33] and aligned using MAFFT v6.240 [34] with the L-INS-I algorithm. To display only a representative and taxonomically diverse but balanced subsample, we selected the human, rat and zebrafish orthologues, except for LGI4 where the missing fish is replaced by dog. Besides the LGI family, only 2 other distinct human proteins contain characteristic EAR domains, C21orf29 and GPR98. To create a multiple alignment of all EAR propeller domains, we selected the respective regions in the sequences from the LGI family (same as used above) and added the EAR regions of C21orf29 and GPR98, from human, rat and zebrafish to maintain the same taxonomic spread for each protein family. Two published reports [3,4] were helpful to reliably identify the sequence borders of each of the individual repeats. Since the structure of the 7 EAR domains is predicted as a seven-bladed beta propeller (1 EAR domain is one blade), we added the human, rat and zebrafish orthologues of WDR5 which is the best predicted propeller template with known structure (see following Methods section). The sequences were aligned with MAFFT v6.240 [34] using the L-INS-I algorithm. The multiple alignment of the EAR propeller domains was rectified, annotated and exported as Additional File 1, Figure S1 with Jalview version 2.3 [35]. NCBI accession numbers of all used sequences are given in the resulting tree figure (Figure 2A). Species are abbreviated as follows: Hs ... Homo sapiens, Rn ... Rattus norvegicus, Dr ... Danio rerio and Cf ... Canis familiaris.

The evolutionary history was inferred using the Neighbor-Joining method [36,37]. The bootstrap consensus tree inferred from 500 replicates [38] is taken to represent the evolutionary history of the taxa analyzed [38]. Branches corresponding to partitions reproduced in less than 50% bootstrap replicates are collapsed. The percentage of replicate trees in which the associated taxa clustered together in the bootstrap test (500 replicates) are shown next to the branches [38]. The tree is drawn to scale, with branch lengths in the same units as those of the evolutionary distances used to infer the phylogenetic tree. The evolutionary distances were computed using the Dayhoff matrix based method [39] and are in the units of the number of amino acid substitutions per site. All positions containing alignment gaps and missing data were eliminated only in pairwise sequence comparisons (Pairwise deletion option). There were a total of 382 positions in the final dataset. Phylogenetic analyses were conducted in MEGA4 [37].

### LGI1 and LGI2 propeller domain structure models

It has been suggested since the first reviews of the LGI family that the C-terminus of these proteins would contain WD40-like repeats forming a beta-propeller [3,4]. State-of-the-art consensus structure prediction methods [40] support this prediction. We find the crystal structure of the beta propeller of WDR5 [PDB:2co0[41]] as highest ranked template for a homology model of the LGI2 propeller domain using the 3D-jury consensus structure prediction server [42]. However, since the sequence identity to this closest template with resolved crystal structure is only roughly 12%, it is important to critically assess the expected reliability of our structural model. To further corroborate our template selection, we show that a direct and significant link can also be established with a simple PSI-BLAST search. Taking the human LGI2 C-terminal domain [UniProt:Q8N0V4 216-545] as query in a PSI-BLAST search (profile inclusion-value 0.001) against UniRef90 (spiked with all non-identical sequences of PDB structures) the WDR5 template structure is found in round 4 with an E-value of 9e-11.

Secondly, the correctness of the sequence alignment is critical. Therefore, our alignment to the template originates from the alignment of representative orthologues of all 4 LGI families as well as other EAR repeat families to WDR5 (see Method section above and Additional File 1, Figure S1) which was then used as input for MODELLER (v9.1) [43] to derive the models for LGI1 and LGI2 (selected from multiple models by the lowest DOPE score). Next, the models were refined (e.g. loops) and energy-minimized through a simulated annealing MD simulation using the AMBER03 force field as implemented in Yasara [44]. All methods used here (from template selection to alignment, modeling and refinement), performed favorably for their respective tasks in the recent community wide experiment on the Critical Assessment of Techniques for Protein Structure Prediction (CASP8).

### Conservation mapping

Orthologues for each LGI subfamily were downloaded from OMA [33] and aligned using MAFFT v6.240 [34] with the L-INS-I algorithm. This alignment was used to calculate the conservation on individual positions using the rate4site algorithm as implemented in the Consurf webserver [45]. The conservation values were mapped to the B-factor column of the PDB file of the structural model (Consurf) and visualized in Yasara as follows: First, the values for all positions are read and normalized. All positions with conservation of at least 90% of the maximal value are classified as highly conserved while those between 70% and 90% are moderately conserved and those below 70% of the maximal value are classified as not conserved. As a difference to classical Consurf and other conservation coloring approaches, we incorporate the physical properties of the amino acids in the color coding. Firstly, all residues that are not conserved are colored grey. Next, moderately and highly conserved residues are colored according to physical properties, i.e., yellow: hydrophobic, green: uncharged polar, blue: positive charge, red: negative charge. The difference between moderately and highly conserved residues (see classification above) is that moderately conserved residues have lower color intensity while highly conserved residues are visualized with full colors. The script for the above described conservation coloring was developed by Joost van Durme, Sebastian Maurer-Stroh and Elmar Krieger and is available in the free version of Yasara http://www.yasara.org.

### SNPs/Mutation analysis

We collected SNP information for LGI2 from NCBI/dbSNP BUILD 129. Four SNPs which cause missense mutations (I24T, K347E, R444Q and Q452R) were found in LGI2. I24T is located near the signal peptide region while the other three SNPs are located in the EAR domains. Besides, we also collected missense mutations of LGI1 which result in ADLTE by reviewing published literature in PubMed. We found 16 known disease missense mutations (C42G, C42R, C46R, A110 D, I122K, E123K, R136W, S145R, L154P, C200R, L232P, I298T, F318C, E383A, V432E and S473L) which are spread over the whole LGI1 sequence (recently reviewed in [10]). We have added the artificial L26R mutation which is located in the signal peptide region of LGI1 for additional study concurrently with LGI2 I24T. Moreover, we constructed two additional artificial mutations with theoretically derived functional hypothesis, LGI1 K353E (corresponds to LGI2 K347E) and LGI2 V420E (corresponds to LGI1 V432E), to test the effect of mutations between identical amino acids at structurally homologous positions in both LGI1 and LGI2 for the wet-lab experiment (see details below).

### Signal cleavage site prediction

Since LGI2 I24T occurs near the signal peptide of LGI2, we investigated whether this mutation has an influence on prediction of the signal peptide and its cleavage site by using the SignalP 3.0 webserver [20]. The first 70 amino acids of either wild-type or I24T LGI2 were used as query sequence. The same procedure was used for prediction of the effect of the artificial L26R mutation in LGI1 which is also located in the respective signal peptide region.

### SIFT and PolyPhen analysis

We investigated which mutations in LGI1 and LGI2 possibly affect function of the proteins by using two prediction methods, SIFT and PolyPhen. In the case of SIFT [46], the LGI family alignment (described above in the part "LGI1 and LGI2 propeller domain structure models"; but using all orthologues rather than the taxonomic subselection) was used. For PolyPhen [19], FASTA sequences of LGI1 and LGI2, respectively, were used as query.

### Expression analysis

Expression data for the 4 LGI genes were retrieved from BioGPS [23], HPRD [24], Human Protein Atlas [25] and SOURCE [26].

### Selecting identical amino acids at structurally homologous positions in both LGI1 and LGI2 for the wet-lab experiment

#### Wet-lab experiments

##### Constructs

LGI1 (NM_005097) ORF construct was purchased from Invitrogen while LGI2 (NM_018176) was purchased from Origene. LGI1 was amplified using forward primer; 5'-CCGCTCGAGCTATGGAATCAGAAAGAAGCAAAAGG-3' and reverse primer; 5'-CGCGTCGACTGCGCTTAAGTCAACTATGAC-3' and cloned into pEGFP-N3 (Clontech). LGI2 was amplified using forward primer; 5'-CCGCTCGAGCTATGGCGCTGCGGAGAGGCGGC-3' and reverse primer; 5'-CCCAAGCTTCCAAACTTAAGTCAACAATTATATG-3' and cloned into pEGFP-N3. Point mutations were introduced into LGI1 and LGI2 using the XL Quikchange site directed mutagenesis kit and verified by sequencing to ensure the integrity of the cloned ORFs. We used all four SNPs of LGI2 and selected four mutations (each from signal peptide region, N-terminal LRR (LRRNT), LRR and EAR), i.e. the artificial L26R (SP), C42R (LRRNT), L154P (LRR) and V432E (EAR) of LGI1 for testing. Besides, we included two additional artificial mutations with theoretically derived functional hypothesis, LGI1 K353E and LGI2 V420E.

##### Cell culture, transfection and media collection

HEK293 cells were grown in Dulbecco's modification of Eagles's medium (DMEM) supplemented with 10% fetal calf serum (FCS) and 1% PSG (penicillin/streptomycin/glutamine) on 6 well plates and maintained at 37°C and 5% CO_2_. HEK293 cells were transiently transfected using Lipofectamine 2000 (Invitrogen). After 17-18 hours of transfection with LGI-GFP constructs in serum free media, the media was collected and centrifuged to pellet cell debris, and the conditioned medium were concentrated using Vivaspin 2 (30000 MWCO PES). Cells were lysed in 3× SDS loading buffer with DTT. Conditioned medium and cell lysate samples were equalized for total protein using the Wako kit.

##### Western blotting

Equivalent volumes of conditioned medium and cell lysates were loaded onto 8% SDS-PAGE gels to resolve proteins. Then, proteins were transferred onto PVDF membrane and blocked using 1% nonfat dry milk for 1 hour to reduce non-specific binding and incubated 1 hour at room temperature with Mouse anti-GFP (Roche) at 1:2500 dilution followed by Goat anti mouse HRP at 1:5000 dilution for 1 hour at room temperature. Immunoblots were developed using ECL Plus (GE healthcare). One set of gel was stained using Simplyblue™safe stain to show that all conditioned media samples were loaded in equal proportions as were the cell lysate samples.

##### PNGase F treatment

HEK293 cells were grown on 10 cm plates and transfected with LGI1 WT and LGI2 WT constructs respectively. Conditioned medium and lysate were collected. The conditioned medium was concentrated using Amicon Ultra-15 30kDa (Millipore) and immunoprecipitated with GFP conjugated beads (Santa Cruz). HEK293 cells were trypsinized and resuspended in HUNT buffer (PBS and complete EDTA free protease inhibitor cocktail tablet) before being subjected to freeze thaw method. Cell lysates were spun down by centrifugation at 12000 rpm for 10 minutes. The supernatant were incubated with GFP conjugated beads at 4°C for 1 hour. The beads were washed five times with HUNT buffer. The immunoprecipitates were denatured using denaturing buffer at 100°C for 10 minutes before being digested by PNGase F (New England Biolabs) at 37°C for 1 hour. The control (-) samples were subjected to the same treatment with PNGase F being replaced with water. The samples were resuspended in 3 × SDS loading buffer for SDS-PAGE.

##### Confocal laser scanning microscopic analysis

After 17-18 hours of transfection with the LGI-GFP constructs using Lipofectamine 2000 (Invitrogen), COS7 cells grown on glass cover slips were fixed with 2% paraformaldehyde in PBS at room temperature. Slides were blocked at room temperature for 1 hour with 5% BSA in 0.3% Triton X/PBS and then immunostained with ER marker anti-PDI (Affinity BioReagents) at 1:1000 dilution followed by Goat anti Mouse Alexa 546 (1: 2000, Invitrogen, Molecular Probes) at room temperature for an hour. Alternatively, COS7 cells were incubated with 10 μM Bodipy TR Ceramide, Golgi marker in HESS/HEPES medium for 30 minutes at 4°C and incubated with fresh HESS/HEPES for 30 minutes before fixing the cells. Images were captured with Zeiss LSM Meta Confocal upright microscopy with a magnification of 63×.

## Abbreviations

LGI: leucine-rich, glioma inactivated; ADLTE: autosomal dominant lateral temporal epilepsy; ADPEAF: autosomal dominant partial epilepsy with auditory features; PEPS: partial epilepsy with pericentral spikes; EPTP: Epitempin; nsSNPs: non-synonymous single nucleotide polymorphisms; LRR: leucine-rich repeat; WT: wild-type; ER: endoplasmic reticulum

## Authors' contributions

VL, FE, SMS designed the computational and LLC, SA the experimental part of the study. Bioinformatics work and literature analysis was done by VL and SMS. The wetlab experiments were executed by LLC, RAABR and SA. The manuscript was written by VL, SA, FE and SMS and was approved by all authors.

## Authors' information

**VL **received a M.Sc. in informational science from Graduate School of Informatics, and D.Sc. from Graduate School of Science, Kyoto University. Meanwhile, she worked as an associate instructor and later as a postdoctoral research fellow in the Bioinformatics Center, Institute for Chemical Research, Kyoto University. Currently, she is a postdoctoral research fellow in the Bioinformatics Institute, A*STAR Singapore.

**LLC **graduated from NUS with a Bachelor of Science degree majoring in Life sciences in 2004. Since then she has been working in research laboratories and has gained experience working on plant, yeast, and mammalian cell systems. Current research interests lie in deciphering the pathways of disease genes.

**RAABR **joined biochemistry and molecular biology lab to do her final year project and she was involved in the experimental part of the project. She has completed her Diploma in Biotechnology, Temasek Polytechnic, Singapore in 2009.

**FE **studied mathematics at 1 the Humboldt-University in Berlin and biophysics and medicine at the Pirogov Medical University in Moscow. He was awarded a MD in 1985. Three years later, he received the PhD in molecular biology from the Engelhardt Institute of Molecular Biology in Moscow (supervision by Dr. Vladimir Gayevich Tumanyan). After postdoctoral work at the Institute of Molecular Biology in Berlin-Buch (1989-1991) and at the EMBL in Heidelberg (1991-1999), he worked as a teamleader of the bioinformatics research group and head of the general IT department at the Institute of Molecular Pathology (IMP) in Vienna (1999-2007). Since August 2007, he is the Director of the Bioinformatics Institute A*STAR Singapore.

**SMS **studied theoretical biochemistry in the group of Peter Schuster at the University of Vienna and wrote his master and PhD thesis while working in Frank Eisenhaber's lab at the Institute of Molecular Pathology (IMP) in Vienna. After a Marie Curie Postdoc fellowship at the VIB-SWITCH lab in Brussels, he joined the A*STAR Bioinformatics Institute (BII) in Singapore where he is heading the Protein Sequence Analysis Group in the Biomolecular Function Discovery Division since 2007. He has contributed widely used predictors for posttranslational modifications and catalyzed new biomolecular insights by sequence-based function predictions.

**SA **was appointed as a Principal Investigator at the Bioinformatics Institute A*STAR Singapore in August 2008. She leads a biochemistry and molecular biology lab that aims to bridge the gap between theoretical predictions of proteins with unknown functions and their cellular biology, which can subsequently be used to aid the identification of novel drug targets. She obtained her PhD degree at the National University of Singapore and worked as a postdoctoral research fellow at Department of Pharmacology, Yong Loo Lin School of Medicine, NUS.

## Supplementary Material

Additional file 1 **The multiple alignment of EAR propeller domains of LGI family, C21orf29 and GPR98**. Human, rat and zebrafish orthologues were selected, except for LGI4 where the missing fish is replaced by dog. Human, rat and zebrafish of WDR5 which is the best predicted propeller template with known structure were also added. The sequences were aligned with MAFFT using the L-INS-I algorithm and the alignment was then rectified, annotated and exported as Figure with Jalview version 2.3. Click here for file
