# Supplementary figures and images for: Similarity of molecular phenotype between known epilepsy gene LGI1 and disease candidate gene LGI2
Source: BMC Biochem. 2010 Sep 24;11:39. doi: 10.1186/1471-2091-11-39 (PMC2949613; doi:10.1186/1471-2091-11-39)

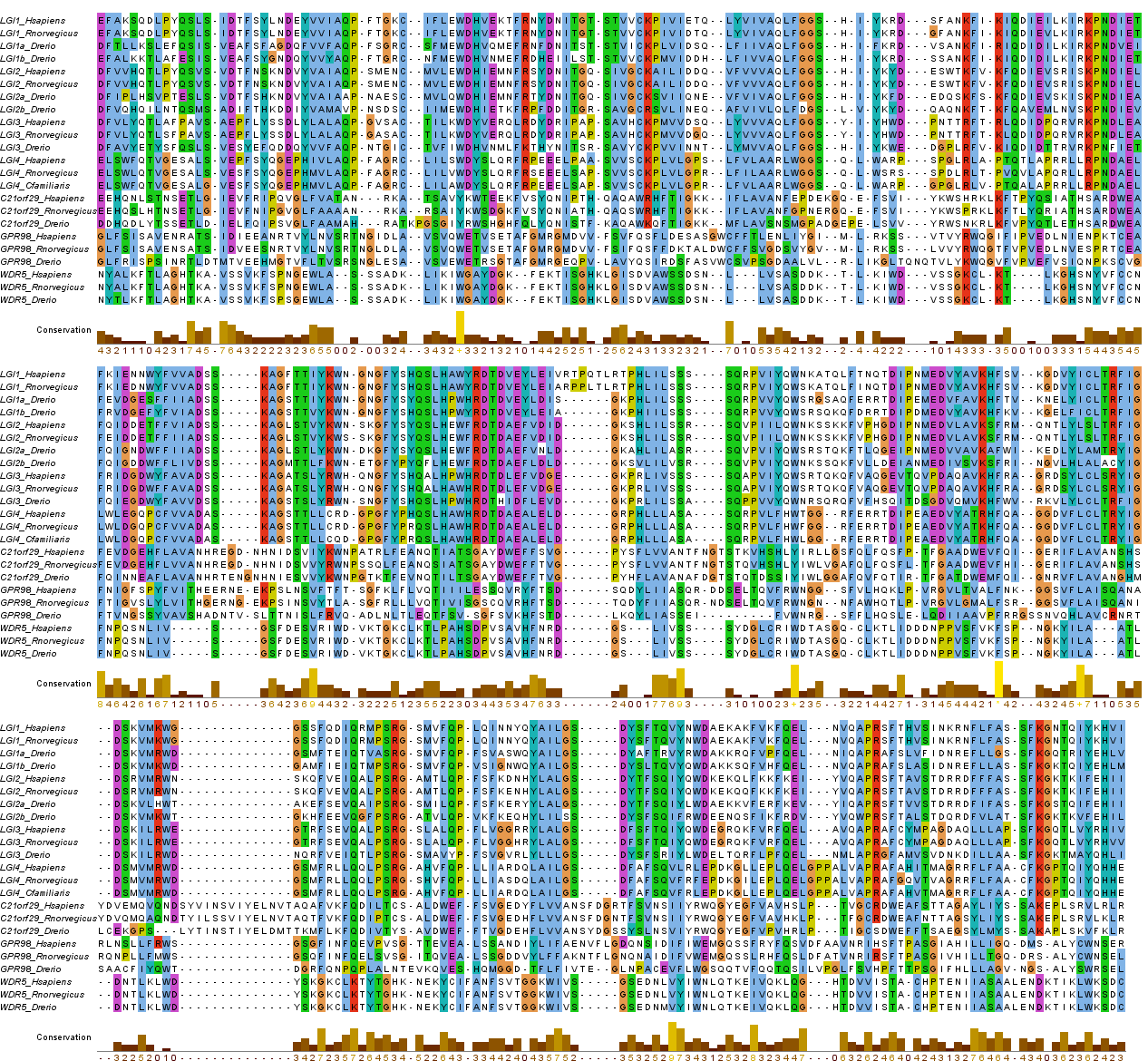

Supplement: Additional file 1 — The multiple alignment of EAR propeller domains of LGI family, C21orf29 and GPR98. Human, rat and zebrafish orthologues were selected, except for LGI4 where the missing fish is replaced by dog. Human, rat and zebrafish of WDR5 which is the best predicted propeller template with known structure were also added. The sequences were aligned with MAFFT using the L-INS-I algorithm and the alignment was then rectified, annotated and exported as Figure with Jalview version 2.3. [file 1471-2091-11-39-S1.PNG]
